# A Lung Segmental Model of Chronic *Pseudomonas* Infection in Sheep

**DOI:** 10.1371/journal.pone.0067677

**Published:** 2013-07-09

**Authors:** David Collie, John Govan, Steven Wright, Elisabeth Thornton, Peter Tennant, Sionagh Smith, Catherine Doherty, Gerry McLachlan

**Affiliations:** 1 The Roslin Institute and Royal (Dick) School of Veterinary Studies, University of Edinburgh, Edinburgh, United Kingdom; 2 University of Edinburgh, Medical School, Edinburgh, Scotland, United Kingdom; University of Duisburg-Essen, Germany

## Abstract

**Background:**

Chronic lung infection with *Pseudomonas aeruginosa* is a major contributor to morbidity, mortality and premature death in cystic fibrosis. A new paradigm for managing such infections is needed, as are relevant and translatable animal models to identify and test concepts. We sought to improve on limitations associated with existing models of infection in small animals through developing a lung segmental model of chronic *Pseudomonas* infection in sheep.

**Methodology/Principal Findings:**

Using local lung instillation of *P. aeruginosa* suspended in agar beads we were able to demonstrate that such infection led to the development of a suppurative, necrotising and pyogranulomatous pneumonia centred on the instilled beads. No overt evidence of organ or systemic compromise was apparent in any animal during the course of infection. Infection persisted in the lungs of individual animals for as long as 66 days after initial instillation. Quantitative microbiology applied to bronchoalveolar lavage fluid derived from infected segments proved an insensitive index of the presence of significant infection in lung tissue (>10^4^ cfu/g).

**Conclusions/Significance:**

The agar bead model of chronic *P. aeruginosa* lung infection in sheep is a relevant platform to investigate both the pathobiology of such infections as well as novel approaches to their diagnosis and therapy. Particular ethical benefits relate to the model in terms of refining existing approaches by compromising a smaller proportion of the lung with infection and facilitating longitudinal assessment by bronchoscopy, and also potentially reducing animal numbers through facilitating within-animal comparisons of differential therapeutic approaches.

## Introduction

With about 80% of CF adults being chronically infected with *Pseudomonas aeruginosa* in the respiratory and conducting zones of the lungs [1] this pathogen is recognized as being a major contributor to morbidity, mortality and premature death in this disease. Patients often become infected with *Pseudomonas* from a young age [2] – with initial isolates resembling those found in the environment – generally non-mucoid strains that are susceptible to antibiotic therapy. With time, these strains adapt to the environment of the CF lung – becoming mucoid, developing biofilms, and becoming relatively resistant to antibiotic therapy [3].

Whilst current treatment for patients with *Pseudomonas* infection revolves around the use of inhaled and systemic antibiotics it is clear that once chronic infection is established no antibiotic is capable of eradicating it. As recently highlighted by Hurley et al (2012) [4] a new paradigm for the management of chronic pulmonary infection with *Pseudomonas aeruginosa* in CF is clearly needed. Whilst new antibiotics that exploit novel mechanisms of action are largely conspicuous by their absence from the current drug development pipeline, the use of adjunct therapies that promote the effectiveness of existing antibiotics hold clinical potential. Such adjuncts include quorum sensing inhibitors, lectin inhibitors and iron chelators – all directed towards controlling the expression of virulence factors, efflux pump modulators, resistance gene expression inhibitors, bacteriophages and endolysins – all directed towards limiting the development of resistance, and lastly immunisation and immunotherapeutic strategies [4].

Whether in the context of antibiotic, gene or adjunct therapies there is wide recognition of the benefits associated with directly targeting the air-tissue interface through aerosol delivery [5]–[7]. As such it is clearly important to study novel therapeutic approaches using animal model systems that are anatomically and physiologically relevant models of the human lung, and that appropriately mimic the chronic aspect of infection whilst permitting the evaluation of therapies in a clinically relevant context.

Our group has extensive experience of modelling the delivery of gene therapy vectors to the healthy respiratory epithelium using the sheep as a model system [8]–[11]. This work, conducted within the framework of the UK CF Gene Therapy Consortium, has culminated in the largest CF clinical gene therapy trial of its kind, currently underway. Our focus on using the sheep in this context was borne out of the realisation that small rodents are a poor model for human lung anatomy and physiology and that this deficiency is magnified when the complexities associated with aerosol delivery are taken into consideration.

The presence of airway pathology associated with chronic infection in cystic fibrosis may add a further barrier to achieving gene expression following gene delivery and it is entirely appropriate both to characterise the likely impact of such pathology on gene delivery and to consider means and methods to circumvent or augment expression in this context. These aims are particularly relevant in the context of reports highlighting the impact of pulmonary infection with *P. aeruginosa* on transfection mediated by viral and nonviral vectors [12], [13]. Clearly the aforementioned limitations of small animal models are equally pertinent in this context and there is a real need to develop alternative models to complement and extend these systems towards developing more effective therapeutic options for *Pseudomonas* infection in the lung.

We present data relating to the characterisation of a novel model of chronic *Pseudomonas* infection in the ovine lung. In line with previous implementations in other species, our strategy involved the suspension of *P. aeruginosa* bacteria in agar beads and the subsequent bronchoscopic instillation of these beads into the distal lung. By tailoring the infective dose we were able to generate a chronic infection in multiple lung segments of the same animals without eliciting any clinical evidence of systemic or respiratory compromise. Presence of infection was maintained for at least two months in individual lung segments. The relationship between infectivity as measured in bronchoalveolar lavage fluid from affected segments and infection present in the same lung segment tissue at necropsy was also explored and highlighted the difficulty of accurately monitoring lung infection *in vivo* – information highly relevant to the design of clinical trials.

## Materials and Methods

### Animals

Ten commercially sourced 10 month-old crossbred lambs (bodyweight: 40.5 kg [35]–[45] Median [range]) were included in this study. Animals were housed for the duration of the study and otherwise maintained according to normal standards of farm animal husbandry. The sheep were treated with anthelminthic before the study began and underwent a preliminary baseline examination involving bronchoscopic visualisation and bronchoalveolar lavage under gaseous anaesthesia immediately prior to treatment to confirm the absence of pre-existing pulmonary disease.

### Experimental design

An initial protocol was designed to characterise the distal lung response to local lung instillation of agar beads alone. Four lung segments in each of two sheep (DR1 & DR2) were instilled with 5 ml agar bead suspension, and the cellular response measured 3, 7, 14 and 21 days later by sampling bronchoalveolar lavage fluid from both an instilled and a control (non-instilled) lung segment. The lungs from these sheep were available for gross examination 84 days after agar bead instillation. Thereafter, following an initial dose-response experiment involving instillations of 1×10^7^, 1×10^8^ and 1×10^9^ cfu *P. aeruginosa* suspended in agar beads, and instillation of agar beads alone, into separate lung segments of the same sheep and evaluation one and three days after infection (DR4) the protocol was extended to compare, again within individual animals, a single lung instillation of 1×10^9^ cfu (in two separate lung segments), and instillation of agar beads alone, with that of a repeated instillation (in a further two segments), with the repeat instillations being given three days after the initial instillation, and evaluation six days after the initial instillation (DR6). This protocol was repeated with an instilled dose 2.5×10^9^ cfu (DR8). The period of evaluation was extended to ten days after the initial instillation in three sheep instilled with either 2×10^9^ cfu (DR11) or 2.5×10^9^ cfu (DR7 & DR19) *P. aeruginosa* cfu. Lastly, the chronicity of infection was investigated in two sheep following a similar protocol but with evaluation 31 (DR12) and 66 (DR18) days after the initial instillation. Details of the protocols, as applied to individual sheep are presented in table 1.

**Table 1:** Details of the experimental protocol as applied to the individual sheep used in the present study.

			Lung segment						
Sheep	Day	Description	RA	RC	RVD2	RCD	LC	LVD2	LCD
DR4	0	Initial instillation		1×10^8^ cfu	1×10^7^ cfu		1×10^9^ cfu	Agarose	
	1	*in vivo* assessment		BBr	BBr		BBr	BBr	BBr
	3	PME		BBr/BAL	BBr/BAL		BBr/BAL	BBr/BAL	BBr/BAL
DR6	0	Initial instillation		1×10^9^ cfu	1×10^9^ cfu		1×10^9^ cfu	1×10^9^ cfu	Agarose
	3	*in vivo* assessment & repeat instillation		1×10^9^ cfu	BAL		BAL	1×10^9^ cfu	
	6	PME		BAL	BAL		BAL	BAL	BAL
DR7	0	Initial instillation		2.5×10^9^ cfu	2.5×10^9^ cfu	2.5×10^9^ cfu	2.5×10^9^ cfu	2.5×10^9^ cfu	2.5×10^9^ cfu
	3	*in vivo* assessment & repeat instillation		2.5×10^9^ cfu	BAL	2.5×10^9^ cfu	BAL	2.5×10^9^ cfu	2.5×10^9^ cfu
	10	PME	BAL	Infl. fixed	BAL	BAL	BAL	BAL	BAL
DR8	0	Initial instillation		2.5×10^9^ cfu	2.5×10^9^ cfu		2.5×10^9^ cfu	2.5×10^9^ cfu	
	3	*in vivo* assessment & repeat instillation		2.5×10^9^ cfu	BAL		BAL	2.5×10^9^ cfu	
	6	PME	BAL	BAL	BAL		BAL	BAL	BAL
DR11	0	Initial instillation		2×10^9^ cfu	2×10^9^ cfu	2×10^9^ cfu	2×10^9^ cfu	2×10^9^ cfu	2×10^9^ cfu
	3	*in vivo* assessment & repeat instillation			BAL/2×10^9^ cfu	2×10^9^ cfu	BAL	2×10^9^ cfu	2×10^9^ cfu
	10	PME	BAL	BAL	Infl. fixed	BAL	BAL	BAL	BAL
DR19	0	Initial instillation		2.5×10^9^ cfu	2.5×10^9^ cfu	2.5×10^9^ cfu	2.5×10^9^ cfu	2.5×10^9^ cfu	2.5×10^9^ cfu
	3	*in vivo* assessment & repeat instillation		2.5×10^9^ cfu	BAL	2.5×10^9^ cfu	BAL	2.5×10^9^ cfu	2.5×10^9^ cfu
	10	PME	BAL	Infl. fixed	BAL	BAL/fixed for EM	BAL	BAL	BAL
DR12	0	Initial instillation		2×10^9^ cfu	2×10^9^ cfu	2×10^9^ cfu	2×10^9^ cfu	2×10^9^ cfu	2×10^9^ cfu
	3	*in vivo* assessment & repeat instillation		2×10^9^ cfu	BAL	2×10^9^ cfu	BAL	2×10^9^ cfu	2×10^9^ cfu
	31	PME	BAL	Infl. fixed	BAL	BAL	BAL	BAL	BAL
DR18	0	Initial instillation		2.5×10^9^ cfu	2.5×10^9^ cfu	2.5×10^9^ cfu	2.5×10^9^ cfu	2.5×10^9^ cfu	2.5×10^9^ cfu
	3	*in vivo* assessment & repeat instillation		2.5×10^9^ cfu	BAL	2.5×10^9^ cfu	BAL	2.5×10^9^ cfu	2.5×10^9^ cfu
	66	PME	BAL	Infl. fixed	BAL	BAL	BAL	BAL	BAL

The lung segment nomenclature is as follows: RA – Right apical; RC – Right cardiac; RVD2 and RCD – Right ventral diaphragmatic 2 and Right caudal diaphragmatic (corresponding to the lateral and dorsal basal segments of the right diaphragmatic lobe respectively); LC – Left cardiac (corresponding to the cardiac segment of the apico-cardiac lobe); LVD2 and LCD – Left ventral diaphragmatic 2 and Left caudal diaphragmatic (corresponding to the lateral and dorsal basal segments of the diaphragmatic lobe respectively). The detailed specification of the lung instillates are as described in the materials and methods. Assessment techniques employed included bronchial brush biopsy (BBr) and bronchoalveolar lavage (BAL). During post mortem examination (PME) some segments were inflation fixed (Infl.fixed) with 10% formaldehyde to facilitate histological assessment, or 3% glutaraldehyde in 0.1 M Sodium cacodylate buffer for electron microscopy.

### Anaesthesia

Food was withheld for 12 hours prior to anaesthesia. General anaesthesia was induced with an intravenous injection of 6–8 mg/kg propofol (Fresenius propofol, 1%, Fresenius Kabi Ltd) and maintained under anaesthesia using positive pressure ventilation (Model 708; Harvard Apparatus, Millis, MA) with a 50∶50 mixture of oxygen and nitrous oxide and 1–3% isoflurane. Tidal volume was adjusted to 10 ml/kg bodyweight and respiratory rate set to maintain end-tidal CO2 in the range 4.5–5.5%.

### Bronchoscopic secretion scoring

A bronchoscope (FG-16X; Pentax, Englewood, CO, USA) was advanced down the trachea and mainstem bronchi and then into the relevant segmental bronchi to enable bronchoscopic scoring according to published method [14].

### Lung instillation

The bronchoscope was advanced towards preselected lung segments and a sterile catheter (OD: 1.8 mm) advanced through the biopsy channel such that it could be visualised in its distal progression to a wedge point and thereafter retracted ∼5 mm in order to avoid forming a seal with the surrounding airway surface. The instilled volume (2.5 ml in all instances) was very gradually introduced into the distal lung segment and followed by a slow bolus of air. Thereafter the bronchoscope was removed from the lung, at all times visualising the airway serving the subtended segment to confirm absence of reflux of instilled material.

### Ethics statement

All experimental protocols were reviewed and approved by the local Roslin Institute ethical review process (The Roslin Institute Animal Welfare and Ethics Committee) and were subject to the provisions of the Animals (Scientific Procedures) Act 1986. During the course of the experimental protocols the animals were assessed on a daily basis for any evidence of adverse effects relating to the procedures involved.

### *P. aeruginosa* culture and bead preparation

*P. aeruginosa* embedded beads were prepared by adapting a published method (12) for use in the sheep model. *P. aeruginosa* mucoid strain PA0579 (a mucoid variant of clinical strain PAO381 isolated using carbenicillin selection [15], supplied by Prof. J. Govan, University of Edinburgh) was grown overnight at 37°C in 20 ml nutrient broth (Oxoid) containing 0.5% yeast extract, then made up to 800 ml and incubated for 4 hours until an OD 600 nm of around 0.8 was reached. Broth culture was centrifuged for 20 minutes at 3000 g then 2 ml of bacterial pellet suspension was mixed with 18 ml molten Tryptic Soy Agarose (15% Tryptone soya broth (Oxoid CM012a), 5% Peptone from soybean meal (Fluka 70178), 5% Sodium chloride, 15% Agarose (Sigma A6877) then steadily injected into rapidly stirring heavy mineral oil (Sigma 330760) pre-warmed to 50°C and containing 0.01% Span 80 (Sigma 09569). Beads were formed by continual stirring for 6 minutes followed by stirring at 4°C for 20 minutes. Mineral oil was removed by centrifugation of beads at 3000 g with 0.5% Sodium deoxycholate (SDC) in PBS then 0.25% SDC in phosphate buffered saline (PBS). Unbound bacteria were removed by centrifugation at 200 g for 5 minutes in sterile PBS. The final loosely packed beads were mixed with an equal volume of sterile PBS and bacterial cfu/ml was calculated by plating out 100 µl dilutions of 1 ml homogenised bead slurry onto *Pseudomonas* Isolation Agar (PIA)(Sigma). Particle sizing of bead preparations (n = 3; LS230, Beckman Coulter, Inc., USA) indicated that bead size averaged 151 µm (SD 104 µm).

### Bronchial brush biopsy

A bronchoscope (FG-16X; Pentax, Englewood, CO, USA) was advanced down the trachea and mainstem bronchi and then into the relevant segmental bronchi until the predefined area selected for brushing was identified. The bronchial brush (BC-202D-2010; Olympus Medical Systems, Tokyo, Japan) was then advanced through the biopsy channel of the bronchoscope, the sheath retracted and the brush applied to the epithelial mucosal surface. By advancing and retracting the brush in contact with the mucosa, a sample of epithelial lining cells was obtained. The bronchial brush biopsy (BBr) sample was vigorously agitated into 1 ml ice cold sterile PBS and stored on ice. A 10 µl sample was removed and checked microscopically for the presence of beads. The remaining sample was homogenised and bacterial load assessed as previously.

### Bronchoalveolar lavage

The bronchoscope was wedged in selected segmental bronchi. Two 20 ml aliquots of PBS (Sigma D8537) were used to collect BALF from selected lung segments. BALF samples were placed into sterile Falcon tubes and immediately placed on ice until subsequent analysis. Five millilitres of BALF was removed and centrifuged at 400 g for seven minutes to separate out the cellular fraction. The resultant pellet was re-suspended in sterile PBS and the total cell number counted before subsequent preparation of cytospins for differential cytology. Cells were counted using a Neubauer haemocytometer and values expressed per millilitre BALF. Cyto-centrifuge slides were prepared and stained using Leishman stain for differential counts on 500 cells. Cells were classified as neutrophils, macrophages, eosinophils, lymphocytes or mast cells according to standard morphological criteria. The remaining BALF was used for assessment of bacterial load.

### Necropsy

Following euthanasia by intravenous injection of baribiturate, the heart and lungs were carefully removed from the carcase following standard necropsy protocols before the heart was dissected away and the lungs presented for further sampling and analysis. The trachea and right and left major bronchi were carefully opened along their dorsal aspect to expose the primary bronchial entrance to each lung segment of interest. Sterile polyethylene tubes were inserted in turn, into each lung segment bronchus until a wedge point was achieved. Thereafter 40 ml sterile PBS (Sigma D8537) was instilled recovered and handled following the same principles employed during bronchoalveolar lavage under anaesthesia. Naïve (no intervention) and control (saline or agar instilled) lung segments were always sampled prior to handling segments previously exposed to *Pseudomonas* and all efforts were directed towards minimising the potential for cross-contamination between lung segments. Lung segments were carefully isolated by gross dissection from surrounding lung tissue before being separately examined and further dissected by parallel transverse sectioning along the plane of the subsegmental bronchus into ∼1cm thick lung slices. Photographic images of the lung slices were collected. Samples were collected for histological evaluation and for assessing the degree of *Pseudomonas* infection. In the latter regard, where gross changes were commonly identified in the segments instilled with *Pseudomonas,* effort was directed towards ensuring that both normal and grossly abnormal tissue was included in samples destined for further evaluation.

### Histopathology

Following formalin fixation lung tissue specimens were paraffin wax embedded and processed following standard protocols and tissue sections prepared and stained with haematoxylin and eosin for qualitative histopathological assessment. In some instances (indicated in table 1) lung segments were inflation fixed with formalin in order to optimise conditions for histomorphological assessment. Detailed histological assessment was applied to lung sections derived from sheep DR4, DR6, DR7, DR11 and DR19, evaluated at ten days, DR12, evaluated at 31 days, and DR18, evaluated sixty-six days after initial lung infection. Sections within sheep were blinded to the assessor.

### Electron microscopy

For transmission electron microscopy, samples were fixed in 3% glutaraldehyde in 0.1 M Sodium Cacodylate buffer, pH 7.3, for 2 hours then washed in three 10 minute changes of 0.1 M Sodium Cacodylate. Following post-fixing in 1% Osmium Tetroxide in 0.1 M Sodium Cacodylate for 45 minutes, the samples were again washed in three 10 minute changes of 0.1 M Sodium Cacodylate buffer. The samples were then dehydrated in 50%, 70%, 90% and 100% normal grade acetones for 10 minutes each, then a further two 10-minute changes in analar acetone before embedding in araldite resin. Sections, 1 μm thick were cut on a Reichert OMU4 ultramicrotome (Leica Microsystems (UK) Ltd, Milton Keynes), stained with Toluidine Blue and viewed in a light microscope to select suitable areas for investigation. Ultrathin sections, 60 nm thick were cut from selected areas, stained in Uranyl Acetate and Lead Citrate then viewed in a Phillips CM120 Transmission electron microscope (FEI UK Ltd, Cambridge, England). Images were taken on a Gatan Orius CCD camera (Gatan UK, Oxon, England).

### FISH

Paraffin wax embedded sections were dewaxed in 2 changes of xylene followed by 2 changes of 100% ethanol, 96% ethanol and three washes in RNase free water. Slides were air-dried then hybridised for 90 minutes at 55°C with *E.coli/P*.*aeruginosa* PNA FISH probe (AdvanDx KT007) using an Hybaid Omnislide in situ hybridisation system. Coverslips were removed and slides briefly washed at 55°C in RNase free water followed by 30 minute wash in wash buffer (AdvandDx). Images were taken with a Nikon EC1 confocal microscope with EZC1 software. Samples of *P. aeruginosa* bacteria air-dried on slides were used as positive controls for the assay.

### *Pseudomonas* infection level

Tissue samples were stored on ice and then finely chopped under sterile conditions. 300 mg of tissue was weighed and placed into Lysing Matrix D tubes (MP Biomedicals 6913–500) containing 600 µl sterile PBS. Samples were homogenised using a Fastprep FP120 (Thermo Electron) with 3 bursts of 20 seconds at setting 6.0 and 5 minute incubation on ice between each homogenisation step. Equal volumes of BALF samples were centrifuged for 10 minutes at 2700 g and each pellet re-suspended in 1 ml sterile ice-cold PBS. BALF pellets and BBr samples were then homogenised as for the tissue samples. Bacterial load was assessed from all samples by preparation of 10 fold dilutions in ice-cold PBS and 100 µl of chosen dilutions spread on PIA plates and incubated overnight at 37°C. Bacterial counts were calculated by manual counting of colony forming units, multiplication by the dilution factor, and a further 10 fold, to allow for 100 µl inoculum to give a final count in cfu/ml.

### Statistical approach

Summary statistics are quoted in the form (Median [range]). Where opportunities to compare paired data sets arose the non-parametric Wilcoxon signed rank procedure was employed. Log transformation was employed as appropriate in order to examine the degree of association and determine the linear relationship between variables (eg BAL cfu/ml, and lesion- and non-lesion cfu) using regression analysis. Spearman's rank order correlation analysis was applied to determining the significance of association between such variables. Statistical software (Minitab 16 Statistical Software, Minitab Inc., State College, PA, USA) and a spreadsheet package (Microsoft Excel, Microsoft, Redmond, Washington) were used in statistical analysis and data visualisation.

## Results

Data from the two sheep subjected to agar instillation alone (DR1 & DR2) indicated that there was virtually no cellular infiltrate as a consequence of instillation. Median [range] total cell counts (×10^6^) in BALF from the agar-instilled segments were 6.27 [4.64–7.90], 5.35 [4.16–6.54], 5.60 [4.02–7.18] and 7.62 [5.74–9.44] at day 3, 7, 14 and 21 after instillation respectively, which compared with values of 4.83 [4.04–5.62], 5.04 [3.60–6.48], 4.93 [4.00–5.86] and 5.34 [3.66–7.02] obtained at the same time from non-instilled control lung segments.

All sheep tolerated the procedures well with no overt evidence of systemic malaise, nor evidence of respiratory compromise such as tachypnoea, dyspnoea or coughing. Appetite was maintained throughout. Six of the eight sheep gained weight during the study (3.5 kg [1]–[9]) whilst two (DR7 and DR8) demonstrated a slight weight loss (−3 and −2 kg respectively, amounting to 7 and 5% of their respective starting weights). There was no significant change in body temperature between baseline measurements (39.2 [38.6–39.6]) and day 3 (39.4 [38.9–39.6]), the time of the second instillation (p = 0.059, Wilcoxon signed rank test). Thereafter low numbers precluded statistical analysis between time points but visual appraisal of the changes in body temperature during the course of each individual protocol indicated that any changes were very minor *i.e.* within 1°C.

**Figure 1:**
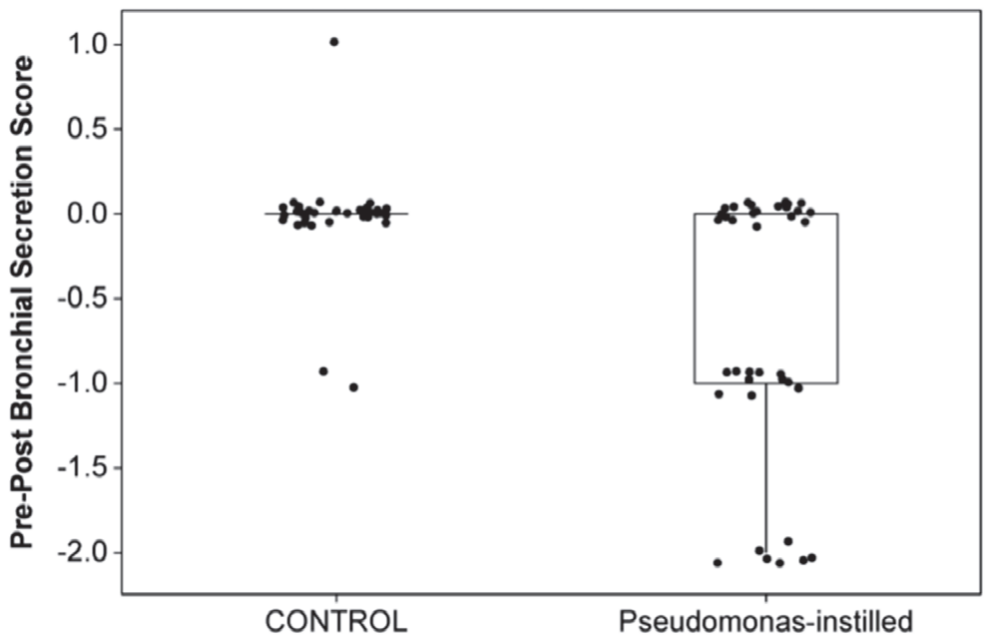
Bronchial secretion scoring. Boxplot illustrating the differences (Pre – Post) in bronchoscopic secretion scores paired by lung segment within sheep and categorised according to whether the segment was instilled with *Pseudomonas* or served as a control (although not included in Table 1, segments RVD1 and LVD1 were also included in the scoring protocol). Instillation with *Pseudomonas* resulted in a small but highly significant increase in bronchial secretion score in the treated segments at day 3 following instillation (d0: 1 (median) [1]–[2] (range) and d3: 1.5 [1]–[3]; p = 0.000, Wilcoxon signed rank test). No significant change in bronchial secretion score was apparent in the control segments (d0: 1 [1]–[2] and d3: 1 [1]–[2]; p = 0.789, Wilcoxon signed rank test).

As the opportunity to examine the trachea and bronchial tree bronchoscopically was afforded immediately prior to instillation and at day 3 following instillation for all sheep, the absence or presence of grossly visible secretions was semi-quantitatively scored by following the scheme of Chang et al (2006) [14]. Figure 1 demonstrates the differences (Pre – Post) in scores paired by lung segment within sheep and categorised according to whether the segment was instilled with *Pseudomonas* or served as a control (although not included in Table 1, segments RVD1 and LVD1 were also included in the scoring protocol). Figure 1 indicates that instillation with *Pseudomonas* results in a small but highly significant increase in bronchial secretion score in the treated segments at day 3 following instillation (d0: 1 [1]–[2] and d3: 1.5 [1]–[3]; p = 0.000, Wilcoxon signed rank test). No significant change in bronchial secretion score was apparent in the control segments (d0: 1 [1]–[2] and d3: 1 [1]–[2]; p = 0.789, Wilcoxon signed rank test).

**Figure 2:**
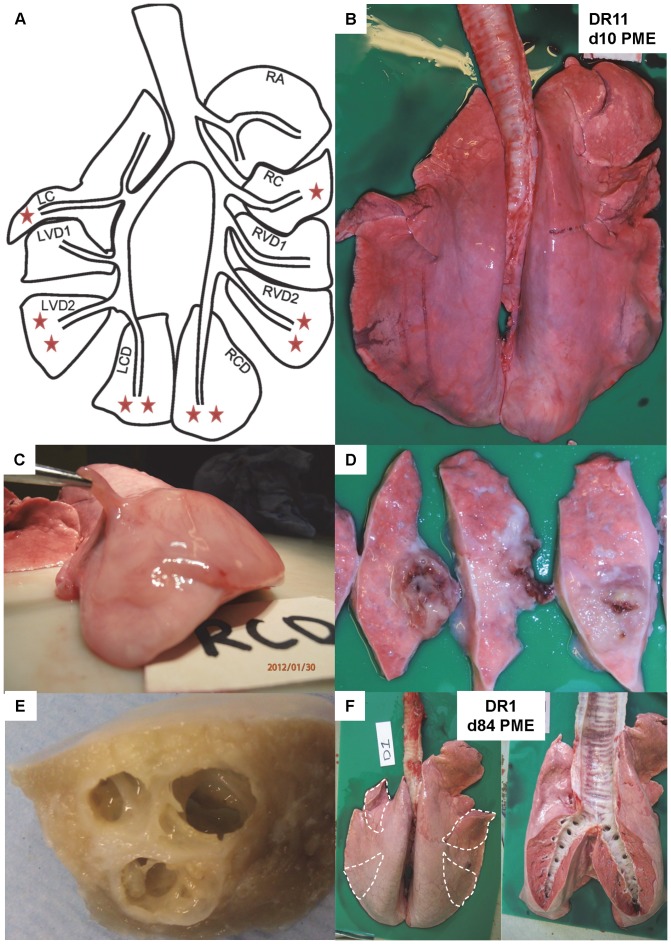
Gross necropsy features of chronic Pseudomonas lung infection in sheep. The lungs of sheep DR11, killed ten days after initial endobronchial instillation with *Pseudomonas aeruginosa*, were broadly indicative of the changes seen in response to infection. Whilst details of the infection protocol are to be found in table 2, the schematic diagram (A) illustrates the anatomical position of the various lung segments together with one or two orange stars to reflect whether a segment received one (on d0, RC and LC) or two (on d0 and d3, RVD2, RCD, LVD2 and LCD) instillations with *P. aeruginosa*. The appearance of the lungs at necropsy (B)(dorsal aspect, heart removed) reflected the instillation schedule with the segments receiving two instillations showing abnormalities in gross appearance. In addition there was evidence of pleural thickening with oedema and fibrosis over segment RCD (C). The cut surface of segment RCD contained areas of haemorrhage and necrosis within forming abscesses (2D). The gross appearance of the cut surface of an inflation fixed lung segment (E)(RVD2) illustrates the nature of these destructive cavitations and their size relative to the cross-sectional area of the segment. The gross appearance of lungs from sheep in which segments (outlined) were instilled with agar beads alone was considered essentially normal at day 84 post instillation (F).

At necropsy examination, it was usually possible to identify treated lung segments on the basis of their visually abnormal appearance (Figure 2B). Typically, infected segments were slightly swollen and had a firmer consistency than surrounding lung and showed variation in colour – sometimes appearing paler, sometimes darker, than the normal lung. In some instances there was evidence of pleural oedema and fibrous tag formation over the affected segment (Figure 2C). In some instances adhesions between the pleural surface and chest wall were apparent. The cut surface of affected segments demonstrated changes that ranged from deep red consolidation to focal areas of firm, pale fibrotic tissue proud of the surrounding lung, to abscesses with variable centres of haemorrhage, necrosis and/or cavitation (Figure 2D, E). Whilst these observations were generally consistent with exposure to bacteria, the co-association was not absolute and in some instances there was little or no gross evidence of pathology to consider. Abnormalities could be appreciated in the gross appearance of some lung segments from DR18, the sheep with the longest duration of infection. In particular there was evidence of pleural oedema and fibrin tag formation overlying and spreading from some of the infected segments. These findings contrasted with the essentially normal gross appearance of the lungs from sheep previously instilled with agar beads alone (DR1 & DR2) (Figure 2F).

Histological appraisal of infected lung segments from sheep DR4 and DR6 (d3 and d6 PME respectively, Table 1) indicated the presence of mild to moderate multifocal pyogranulomatous and suppurative pneumonia typically centred on eosinophilic agar beads. The beads were usually superimposed with granular to slightly filamentous basophilic material (presumed bacterial microcolonies). Additional findings in infected segments concerned the presence of moderate multifocal bronchial associated lymphoid tissue (BALT) hyperplasia and focal haemorrhage. Mild to moderate BALT hyperplasia and patchy atelectasis was noted in the lung segments instilled with agar beads alone.

**Figure 3:**
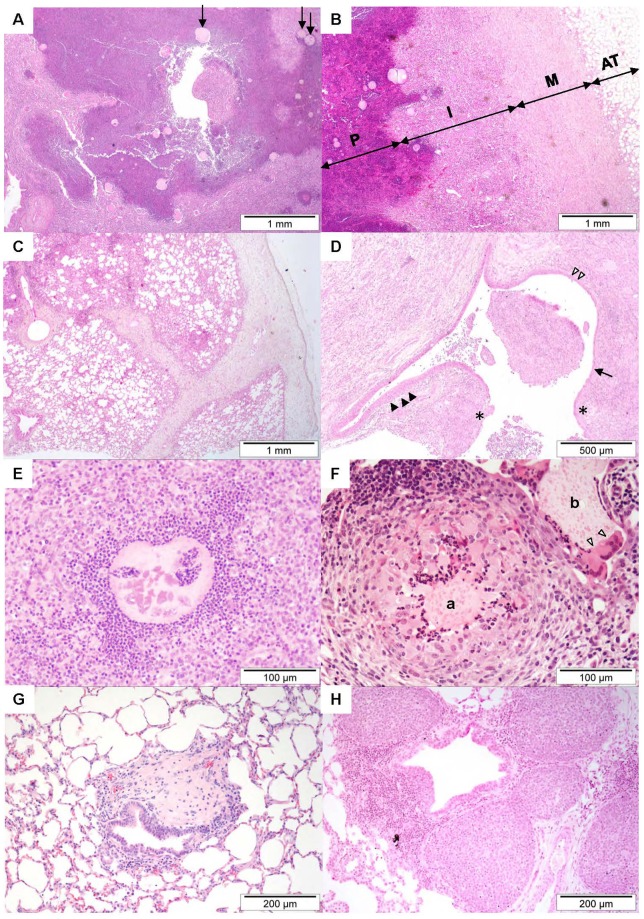
Histopathological features of chronic Pseudomonas lung infection in sheep. Images from histological sections that exemplify the histopathological features of chronic *P.aeruginosa* lung infection in sheep. All sections were stained with haematoxylin and eosin. (A) Low power view of a histological section derived from an inflation-fixed lung segment ten days after initial infection with *P.aeruginosa* (DR7, segment RC). There is large scale obliteration of normal distal lung architecture by a fairly well-defined expanse of necrotic and viable neutrophils surrounded in turn by macrophages (pyogranulomatous inflammation). Individual beads are readily recognised (arrows). (B) In this low power micrograph image from a neighbouring field to that presented in (A) the pyogranulomatous core (P) replaces normal alveolar structure and is bordered by an innermost layer of immature granulation tissue (I) and an outer layer of mature granulation tissue (M). The layers of granulation tissue obscure and replace the normal pleura and extend to adjacent adipose tissue adhered to the pleural surface (AT). (C) Low power micrograph image (DR7, segment LCD) illustrating the marked interlobular septal and pleural fibrosis that characterises the chronic response to *P.aeruginosa* lung infection in sheep. (D) Low power micrograph image centred on a cavity (DR11, RVD2; see Figure 2E for gross appearance). The boundary of the cavity variably consists of granulation tissue (*) and mucosal epithelium. The inflamed granulation tissue consisted of fibroplasia, fibrosis and superimposed neovascularisation, infiltrated by numerous clusters of neutrophils and epithelioid macrophages with formation of pyogranulomas and many entrapped beads. The mucosal epithelium ranges from respiratory (ciliated pseudostratified with goblet cells) (▴) to cuboidal (▵) or attenuated (↑). (E) Micrograph (DR19, LVD2) illustrating the typical appearance of a bead surrounded by neutrophils, some of which infiltrate the bead substance. The amorphous eosinophilic material within the bead is interpreted as *P. aeruginosa* microcolonies. (F) Micrograph (DR12, RC) from an inflation fixed lung segment that shows two beads (a, b). Whilst both beads are cuffed by neutrophils, bead b is also closely associated with some multinucleated giant cells (▵). (G) Micrograph (DR18, LC) from a lung segment infected with *P.aeruginosa* 66 days previously. A discrete localised island of mature fibrosis lies adjacent to a bronchiole. (H) Micrograph (DR11, RA) from a lung segment not exposed to any intervention. There is extensive BALT hyperplasia with formation of germinal centres surrounding a bronchiole.

Histological appraisal of lung segments from sheep DR7, DR11 and DR19 (d10 PME, Table 1) indicated that Pseudomonas bead instillation was associated with the development of a severe, multifocal, pyogranulomatous, suppurative and necrotising pneumonia centred on the instilled beads. To exemplify, changes present in segment RC from sheep DR7 were characterised by the large scale obliteration of the alveolar spaces by multifocal, sometimes coalescing or cavitated, variably sized pyogranulomas which consisted of large central cores of necrotic and viable neutrophils mixed with granular eosinophilic cell debris and surrounded by moderate to large numbers of macrophages, with fewer lymphocytes and plasma cells (Figure 3a). The macrophages ranged from finely vacuolated to epithelioid, the relative proportions tending to vary with area examined. Some pyogranulomas were discrete but there was also more expansive effacement of lung parenchyma. The pyogranulomas were often bordered by granulation tissue and fibrosis (Figure 3b) which, in some areas, obscured the border between lung parenchyma and pleura, effaced the pleura and extended to adjacent extra-pulmonary (pericardial) fat. The granulation tissue also blended with anastomosing trabeculae of slightly oedematous fibrous connective tissue which accentuated the lung lobules. Agar beads could be identified scattered throughout, most notably within the pyogranulomas, and some of these structures were fractured, contained numerous colonies of bacteria and were rimmed or invaded by neutrophils and/or macrophages (Figure 3e). Within the inflamed areas, some bronchioles were lined by increased numbers of epithelial cells, indicative of hyperplasia. The few recognisable alveolar spaces which remained contained oedema and fibrin and there were also peripheral areas of haemorrhage. In similarly treated lung segments from the same sheep (RCD, LVD2 and LCD) there were changes consistent with these observations with, in addition, interlobular septal fibrosis and oedema that extended to the pleural surface (Figure 3c). In LVD2 the pleura was also thickened by granulation tissue. Changes within similarly treated segments of sheep DR11 and DR19 were broadly consistent with the above with, in addition, patchy thickening of the alveolar walls by macrophages and plump lining epithelial cells, compatible with type II pneumocyte hypertrophy (LVD2, DR11) and follicular expansion of BALT surrounding bronchioles. Whilst histological features of control segment RA from DR19 were within normal limits, BALT hyperplasia with formation of germinal centres was a feature of the control segment (RA) from sheep DR11 (Figure 3h), as were interlobular septal oedema and lymphangiectasia. In the control segment (RA) from sheep DR7 there was also interlobular septal oedema which extended into the pleura, accompanied by neovascularisation and small numbers of lymphocytes, plasma cells and neutrophils. The pleura of this segment was covered by a thick layer of fibrin admixed with more numerous neutrophils and macrophages. The pleural mesothelial cell layer was either effaced or the cells that remained were hypertrophic. The subpleural parenchyma featured a band of type II pneumocyte hypertrophy. Randomly scattered and more discrete pyogranulomas were also a central feature of the changes present at day 31 in segments treated with Pseudomonas (DR12). These pyogranulomas were largely characterised by tight nests of epithelioid macrophages swirling around central clusters of neutrophils. Epithelioid macrophages were the predominant cell type present whereas neutrophils were present in much lower numbers relative to the changes seen at day 10. Beads were identifiable in the parenchyma but also in the bronchioles where they were often surrounded or infiltrated by small numbers of neutrophils and multinucleated giant cells (Figure 3f). Some of the granulomas were circumscribed by circumferential rings of fibroblasts and collagen, consistent with fibroplasia and fibrosis. BALT hyperplasia also featured as did interlobular septal fibrosis which extended from the pleural surface. The fibrosis was also a significant feature of segment RVD2 (challenged on one occasion with Pseudomonas). In this segment there was marked, multifocal perivascular, septal and pleural fibrosis, with broad tags of loose fibrous connective tissue extending out from the pleural surface. In the worst affected areas, there was moderate thickening of the alveolar walls by an increased amount of collagen, with almost complete obliteration of alveolar spaces. Histological features of the control segment (RA) from sheep DR12 were within normal limits. Histopathological features of the infected lung segments of DR18, the sheep in which infection had persisted for 66 days, were largely consistent with the features seen at the other time points. However, notable features were the predominance of lymphocytes over neutrophils and plasma cells in sections, the presence of fibrous connective tissue in the interlobular septa and pleura, the presence of discrete localised areas of mature fibrosis often adjacent to bronchioles (Figure 3g), BALT hyperplasia, mineralisation of some bead structures and the presence in inflammatory infiltrates of haemosiderophages. In one sheep (DR11, RVD2) the lung parenchyma contained three coalescing cavities measuring from 5 to 8 mm in diameter and bordered mainly by inflamed granulation tissue but also segmentally by mucosal epithelium. The inflamed granulation tissue consisted of fibroplasia, fibrosis and superimposed neovascularisation, infiltrated by numerous clusters of neutrophils and epithelioid macrophages with formation of pyogranulomas and many entrapped beads. The mucosal epithelium ranged from respiratory (ciliated pseudostratified with goblet cells) to cuboidal or attenuated (Figure 3d). The lumen of these cavities was either empty of partially filled with inflammatory cells, as described above.

**Figure 4:**
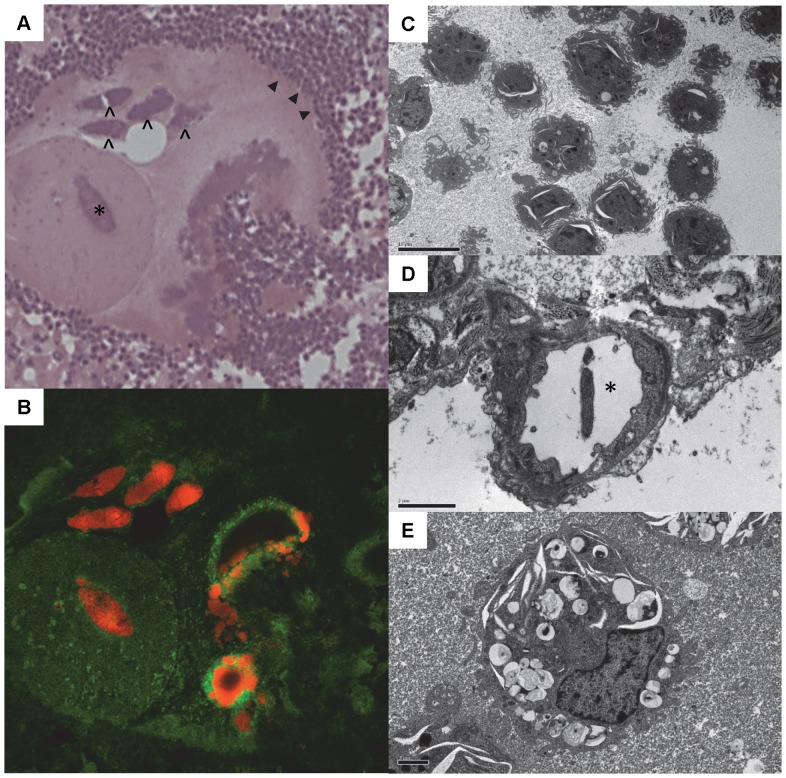
Peptide nucleic acid fluorescence in situ hybridization (PNA FISH) and transmission electron micrography (TEM) of infected lung tissue. Serial sections from an infected segment (DR12, LCD) confirming the identity of the presumed bacterial microcolonies growing in the agar beads. The haematoxylin and eosin stained section (a) has at its centre, a discrete circular bead containing one presumed cigar-shaped bacterial colony (*), and partially surrounded by another predominantly circular bead structure containing a further four distinct presumed colonies (∧). The peripheral margin of the coalesced bead matrix is surrounded by a halo of neutrophils (▴) which appears to infiltrate towards the centre from the lower right quadrant. The PNA FISH image (B) is from a section serial to (A), and confirms that the presumed colonies are indeed made up of *Pseudomonas aeruginosa* bacteria (bright orange stain). TEM images confirmed that the predominant cell type surrounding beads was the neutrophil. In many instances these cells had a highly ruffled surface architecture of microvillus-like projections (C) (bar  = 10 µm). Only occasionally were single bacteria found separate from the agar bead matrix environment, and occasionally found in blood or lymphatic vasculature (D, *) (bar  = 2 µm). Macrophages showed evidence of bacterial engulfment (E) (bar  = 2 µm).

The FISH technique confirmed the identity of the presumptive bacteria present in the bead substance. Evidence of colonisation appeared to be restricted to the beads (Figure 4a, b). Whilst electron microscopic characterisation of *Pseudomonas* microcolonies in the agar beads proved elusive, bacteria were observed occasionally in isolation, and frequently following engulfment by phagocytic cells (Figure 4c–e).

Data from the two sheep subjected to agar instillation alone (DR1 & DR2) indicated that there was virtually no cellular infiltrate as a consequence of instillation. Median [range] total cell counts (×10^6^) in BALF from the agar-instilled segments in these sheep were 6.27 [4.64–7.90], 5.35 [4.16–6.54], 5.60 [4.02–7.18] and 7.62 [5.74–9.44] at day 3, 7, 14 and 21 after instillation, which compared with values of 4.83 [4.04–5.62], 5.04 [3.60–6.48], 4.93 [4.00–5.86] and 5.34 [3.66–7.02] obtained at the same time points from non-instilled control lung segments. In sheep DR4, segment LVD2 was instilled with agar beads alone and the total BALF cell count 3 days after instillation was 0.34×10^6^. Similarly in sheep DR6, segment LCD was instilled with agar beads alone and the total BALF cell count 6 days after instillation was 6.34×10^6^.

**Figure 5:**
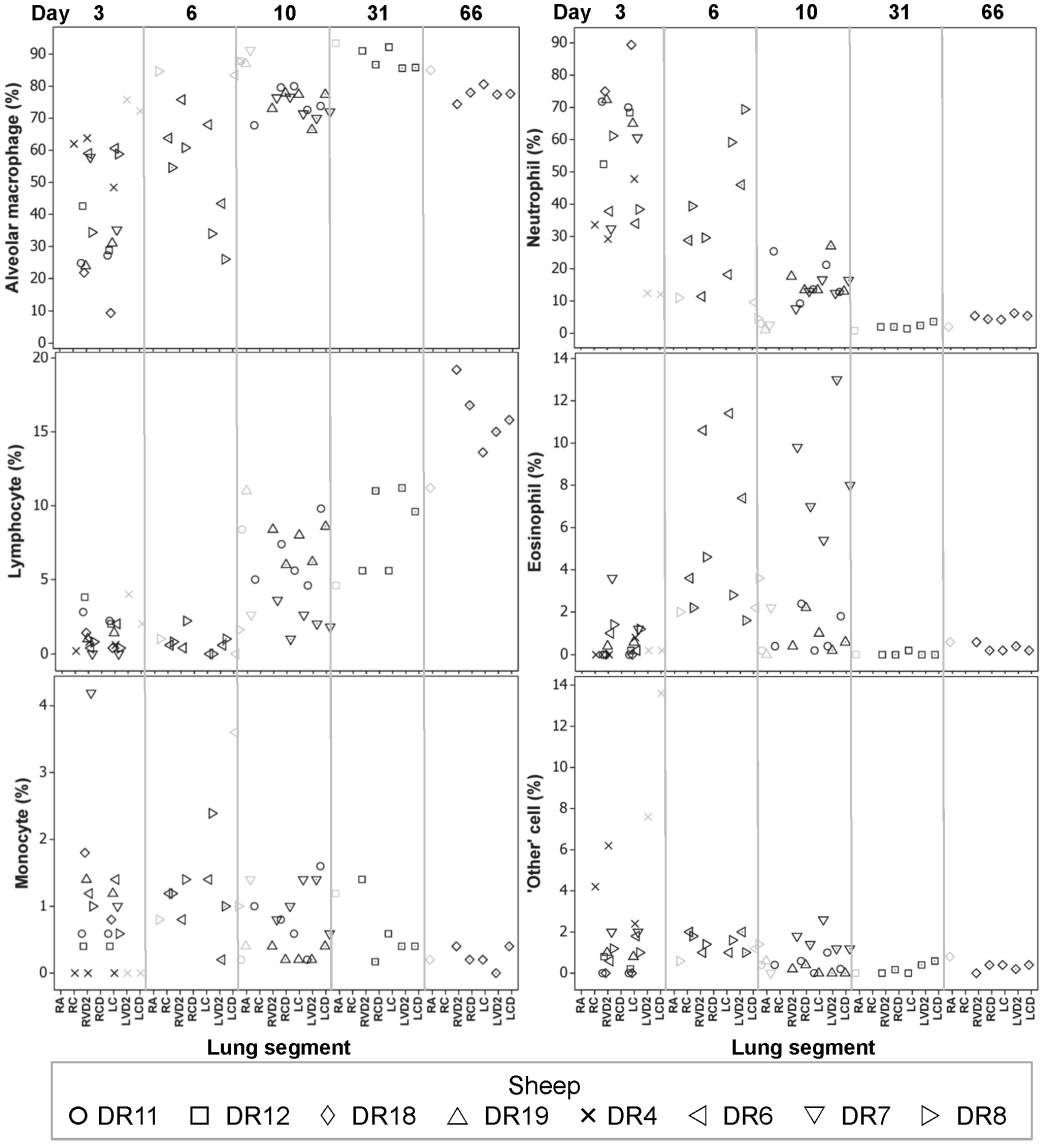
Differential cytology of bronchoalveolar fluid samples. Plots illustrating the relative proportions (%) of cells found in the bronchoalveolar lavage fluid samples, ordered according to the day after the first lung instillation (category specified at the top of the graph columns) on which the sample was derived, and then according to the lung segment from which the sample was derived (categorised on the x-axis). The symbols relate to the sheep from which the samples originate and can be identified from the legend. Additional information of relevance to interpreting the graphs relates to whether the samples are derived from a control segment, in which case the symbol is faint, or whether the samples are derived from a segment subject to two instillations of *P. aeruginosa* (at d0 and d3) rather than one, in which case the double-dosed segments are identified by a dot placed within the symbol eg ◊ reflects the single- and 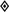 the double-dosed segments from DR18.

The median total cell count (TCC) present in BAL fluid from the 8 control lung segments (not subject to any instillation) sampled at PME was 7.8×10^6^ (range 0.2–18.7) and mostly comprised alveolar macrophages (87% [72–93]), lymphocytes (4% [1]–[11]) and neutrophils (3% [1]–[12]). In contrast, after one lung segmental instillation of *Pseudomonas* the median TCC (×10^6^) was 16.5 (range 0.4–117.3) at 3 days, 16.3 (7.7–50.2) at 6 days, 28.6 (14.2–48.4) at 10 days, 11.5 (6.8–16.2) at 31 days and 10.2 (8.0–12.3) at 66 days. After two lung segmental instillations of *Pseudomonas* the median TCC was 130.5 (37.8–178.7) at 6 days, 41.5 (18.7–86.0) at 10 days, 11.8 (11.3–14.4) at 31 days and 11.0 (8.5–13.8) at 66 days. The change in the relative proportion of cells comprising the TCC is depicted in Figure 5 and illustrates that the increase in TCC 3, 6 and 10 days after instillation is accompanied by an increase in the proportion of neutrophils and a mirrored fall in the proportion of alveolar macrophages. Another notable feature of the differential cell response was the apparent trend towards an increase in the proportion of lymphocytes at d10 (D7, D11 & D19), d31 (D12) and d66 (D18).

**Figure 6:**
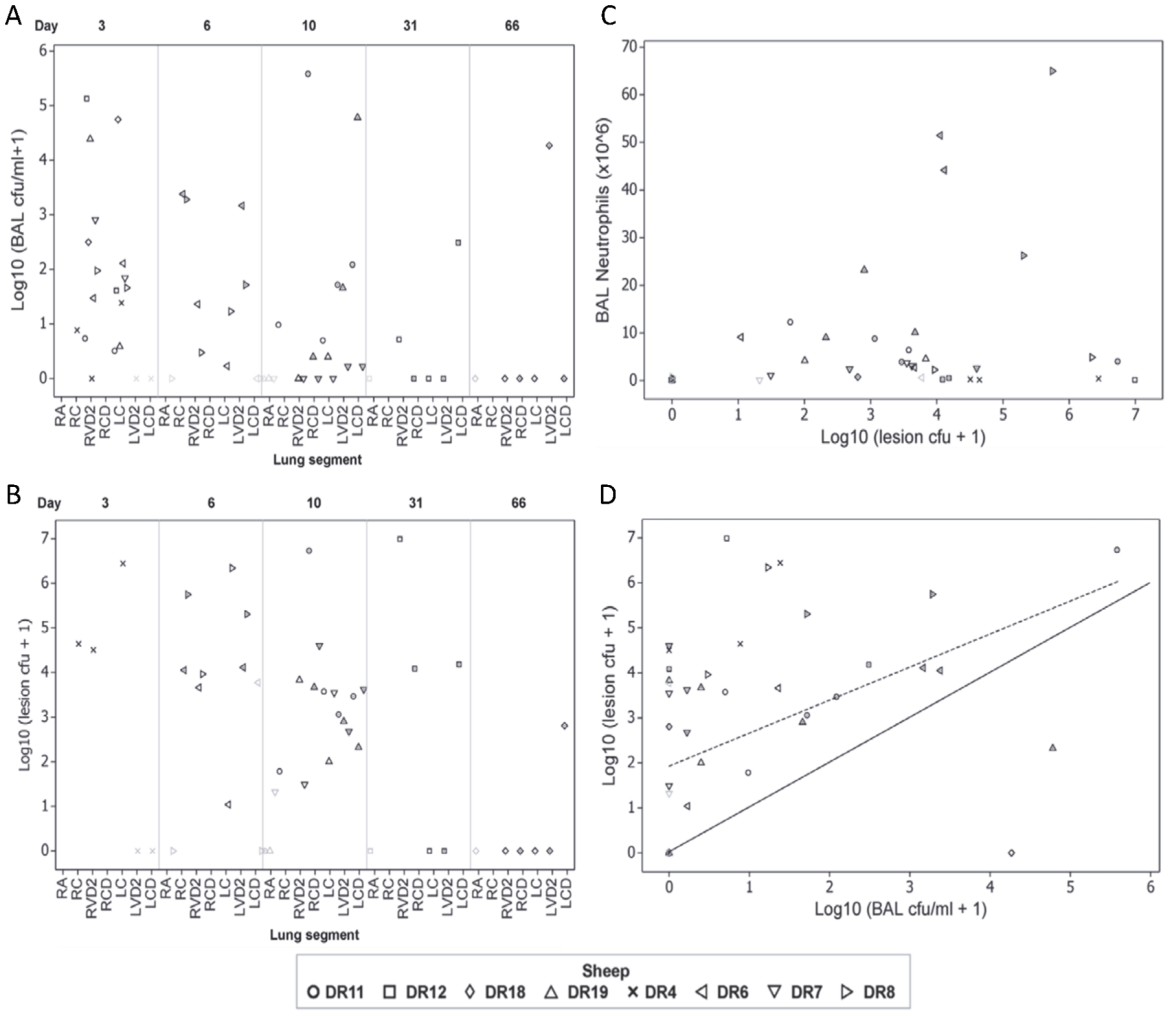
A. The burden of infection in bronchoalveolar fluid samples. Plot of the burden of infection found in bronchoalveolar lavage fluid samples (Log_10_(BAL cfu/ml +1)) expressed in relation to time (Day) after the initial infection (category specified at the top of the graph) on which the sample was derived. The x-axis is further categorised according to the lung segment from which the sample was derived. See legend for Figure 6 for an explanation of the further legend categorisation applied at the level of individual symbols. B. The burden of infection in lung tissue samples. Plot of the burden of infection found in lesion tissue samples (Log_10_(lesion cfu +1)) expressed in relation to time (Day) after the initial infection (category specified at the top of the graph) on which the sample was derived. C. The association between the burden of infection and inflammation. Absolute neutrophil counts (BAL Neutrophils (×10^6^)) in bronchoalveolar lavage fluid samples is plotted against the burden of infection (Log_10_(lesion cfu +1)) in lesion tissue derived from the same segments. The symbols relate to the sheep from which the samples originate and can be identified from the legend. Additional information of relevance to interpreting the graphs relates to whether the samples are derived from a control segment, in which case the symbol is faint, or whether the samples are derived from a segment subject to two instillations of *Pseudomonas* aeruginosa (at d0 and d3) rather than one, in which case the double-dosed segments are identified by a dot placed within the symbol eg ◊ reflects the single- and 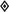 the double-dosed segments from DR18. D. The association between the burden of infection in lung tissue and bronchoalveolar lavage fluid derived from that tissue. Scatterplot illustrating the nature of the association between the burden of infection found in lesion lung tissue (Log_10_(lesion cfu +1)) and that found in bronchoalveolar lavage fluid (Log_10_(BAL cfu/ml +1)) sampled from the same segment. Data from control segments is also shown for comparison (faint symbols). A highly significant positive correlation (*r*_s_ = 0.574, p = 0.000) exists between the variables. The solid line is the line of identity, and the dotted line indicates the linear relationship between these variables – described by a linear relationship of the form Log_10_(lesion cfu +1)  = 1.895+0.679× Log_10_(BAL cfu/ml +1).

*Pseudomonas* infection level: In one sheep (DR4) bronchial brush biopsy samples were collected at day 1 and day 3 after initial instillation in order to ascertain whether *Pseudomonas* infection could be detected in those samples. We were unable to detect any evidence of infection (data not shown) and this method of sampling was discontinued thereafter. The results of dilution analysis to determine the level of infection present in BAL fluid and in tissue homogenates from lung segments are presented in Table 2. Figure 6A plots the Log_10_(BAL cfu/ml +1) data against time (BAL Day), and further categorises this data on the basis of lung segment, sheep identity and segmental protocol (control, single and double instillation). The time-dependent resolution of the initial infection is apparent in BAL fluid derived from previously instilled lung segments. Levels ranged from no evidence of infection through to 3.8×10^5^ cfu/ml of BAL fluid (DR11, RCD, d10). With one exception (DR4, RVD2) BAL fluid derived from previously infected lung segments contained demonstrable levels of infection at d3 and d6 after infection. At d6 after infection, those lung segments that received an infective dose on two occasions (d0 and d3) had increased levels of infection present in BAL fluid relative to those that only received one infective dose (d0). By day 10, although the highest levels of infection were found in BAL fluid derived from segments that received an infective dose on two occasions, some segments that were similarly dosed had little (DR19, RCD; DR7, LVD2 and DR7, LCD) or no (DR7, RCD) evidence of infection by this time point. Levels of infection were still demonstrable in BAL fluid derived from some lung segments 31 and 66 days after infection, with no infection demonstrable in the remainder. No evidence of infection was present in BAL fluid derived from control lung segments.

**Table 2:** Counts of colony forming units (cfu) present in samples derived from the bronchoalveolar space (BAL – Bronchoalveolar lavage fluid) and from lung tissue dissected at necropsy.

		Lung Segment																				
		RA			RC			RVD2			RCD			LC			LVD2			LCD		
		Tissue			Tissue			Tissue			Tissue			Tissue			Tissue			Tissue		
Sheep	Day	B AL	Les ion	Non -lesion	BAL	Les ion	Non- lesion	BAL	Les ion	Non- lesion	BAL	Les ion	Non- lesion	BAL	Lesi on	Non-lesion	BAL	Les ion	Non- lesion	BAL	Les ion	Non- lesion
DR4	3				7	4.4×10^4^	70	0	3.2×10^4^	150				23	2.8×10^6^	2.0×10^3^	0	0	0	0	0	0
DR6	3							29						128								
	6				2.4×10^4^	1.1×10^4^	50	22	4.6×10^3^	1.3×10^3^				1	10	1.3×10^3^	1.5×10^3^	1.3×10^4^	2.7×10^3^	0	5.9×10^3^	2.2×10^3^
DR7	3							808						68								
	10	0	20	50				0	30	30	0	4.0×10^4^	60	0	3.5×10^3^	0	0.67	480	160	1	4.2×10^3^	20
DR8	3							93						45								
	6	0	0	20	1.9×10^3^	5.6×10^5^	830	2	9200	110				16	2.2×10^6^	160	51	2.0×10^5^	860	0	0	10
DR11	3							4						2								
	10	0	0	0	9	60	190				3.8×10^5^	5.4×10^6^	3.4×10^6^	4	3.8×10^3^	30	51.33	1.2×10^3^	20	121	2.9×10^3^	110
DR19	3							2.4×10^4^						3								
	10	0	0	0				0	6.8×10^3^	0	2	4.7×10^3^	320	2	100	20	45	800	280	6.1×10^4^	210	1.6×10^4^
DR12	3							1.3×10^5^						40								
	31	0	0	0				4	9.8×10^6^	2.8×10^4^	0	1.2×10^4^	1.3×10^4^	0	0	0	0	0	0	307	1.5×10^4^	510
DR18	3							312.86						5.6×10^4^								
	66	0	0	0				0	0	0	0	0	0	0	0	0	1.9×10^4^	0	0	0	640	0

Counts of colony forming units (cfu) present in samples derived from the bronchoalveolar space (BAL – Bronchoalveolar lavage fluid) and from lung tissue dissected at necropsy from lung segments subjected to the treatment protocols as specified in table 1. Where the cut surface of lung segments demonstrated areas of gross abnormality, then these areas were specifically sampled and designated in the table as “Lesion”. “Non-lesion” lung tissue had no gross evidence of abnormality. Bronchoalveolar cfu counts are expressed per millilitre of recovered bronchoalveolar lavage fluid, and tissue cfu counts are expressed per gram of tissue.

Our necropsy protocol involved the separate consideration of grossly normal (non-lesion) and grossly abnormal (lesion) tissue from lung segments and we were able to ascertain the levels of infection present in these paired sample sets (Table 2). Linear regression analysis of the tissue infection level data indicated a strong positive correlation between Log_10_(lesion cfu +1) and Log_10_ (non-lesion cfu +1) (r^2^  = 0.75; p = 0.000, Pearson correlation) with the relationship described by Log_10_(lesion cfu +1)  = 0.781 +1.07×Log_10_ (non-lesion cfu +1) indicating that levels of infection measured in non-lesion tissue surrounding areas of gross pathology in this model are approximately six-fold less than levels found within the areas of gross pathology. Figure 6B [lesion infectivity vs time] plots the Log_10_(lesion cfu +1) data against time (Post mortem examination (PME) Day), and further categorises this data on the basis of lung segment, sheep identity and segmental protocol (control, single and double instillation). Of note is the apparent presence of infection in four control or agar-only instilled lung segments from three sheep (DR6:LCD, DR7:RA, DR8:RA and DR8:LCD).

Spearman's rank order correlation analysis was applied to determining the significance of association between Log_10_(lesion cfu +1) data and absolute cell counts present in BAL fluid derived from the same segments at the time of PME. This analysis determined that there was a significant positive correlation between Log_10_(lesion cfu +1) and absolute counts of neutrophils (Spearmans rho (*r*_s_)  = 0.462, p = 0.001), eosinophils (*r*_s_ = 0.321, p = 0.025), monocytes (*r*_s_ = 0.379, p = 0.007) and ‘other’ cells (*r*_s_ = 0.426, p = 0.002). The association between Log_10_(lesion cfu +1) and absolute neutrophil counts in BAL fluid is depicted in Figure 6C [Lesion infectivity vs PMNs].

Correlation between Log_10_(lesion cfu +1) and Log_10_(BAL cfu/ml +1) data demonstrated a highly significant positive correlation (*r*_s_ = 0.574, p = 0.000). The nature of this association, described by a linear relationship of the form Log_10_(lesion cfu +1)  = 1.895+0.679× Log_10_(BAL cfu/ml +1), is depicted in Figure 6D.

## Discussion

Regional bronchoscopic instillation has been extensively used to model lung infection in large animals and such applications have included modelling *Mycobacterium tuberculosis* Infection [16], [17] and group A *Streptococcal* bronchopneumonia [18] in Cynomolgus Macaques, *M.bovis* infection in rabbits [19], respiratory *Chlamydia psittaci* infection in calves [20], and *Mannheimia haemolytica* [21], [22] and *Pseudomonas* [23] pneumonia in sheep. In the last regard Patterson and Todd (1982) [23] described a model in which between 5.0×10^4^ and 12.4×10^9^ *Pseudomonas* organisms were instilled in bronchi – a technique which was repeated 24 hours later. The sheep were killed 3 days after the first instillation. *Pseudomonas*-instilled sheep developed a persistent cough, hypoxaemia and pulmonary hypertension associated with the development of an acute haemorrhagic lobar pneumonia characterised at the histological level by extensive polymorphonuclear infiltrates, thickened alveolar septa and haemorrhagic consolidation.

One of the earliest described animal models of chronic *Pseudomonas* infection was that of Cash et al (1979) who embedded *P. aeruginosa* in agar beads and inoculated these beads into the lungs of rats via the intratracheal route [24]. The inoculation resulted in a chronic non-lethal infection that resembled the lesions seen in the lungs of humans with acute or chronic P.*aeruginosa* infection. This model system [25]–[28] and its adaptation to mice [29], [30] remains a popular choice to this day and has also been employed in cats [31], [32] and rhesus monkeys [33], [34].

Our results indicate that there is minimal measurable response, in terms of total bronchoalveolar cell infiltrates, to the instillation of agar beads alone. All values and ranges for segments instilled with agar alone lay comfortably within the observed range of values for total cell count for control lung segments not subject to any instillation, and are within the expected range for total bronchoalveolar cell counts in healthy sheep in our laboratory (D Collie, personal communication). Similarly, histopathology associated with bead instillation was (SS)??, and the outward appearance of the lungs from sheep instilled with agar beads 12 weeks previously was essentially normal with none of the gross abnormalities associated with *P.aer* infection being observed. Our observations are in line with data from the original rat model system. In this model Cash et al (1979) demonstrated that agar beads alone prompted only a mild parabronchial inflammatory response that usually resolved within one week [24]. Similarly, again using the rat model, Growcott et al (2011) found that sterile agar beads elicited no change in BALF cellularity between day 2 and day 7 after instillation [26].

In contrast, distal deposition of *P. aeruginosa* in agar beads was associated with the development of severe, multifocal, suppurative, necrotising and pyogranulomatous pneumonia centred on the instilled beads. It is appropriate to consider whether the response to bead instillation bears resemblance to that described for *Pseudomonas* infections of the human lung. The hallmarks of *Pseudomonas* infections in the human lung are haemorrhage, necrosis and abscess formation. Such infections generally arise either as a consequence of aspiration via the airways or via bacteraemic spread. Microscopically the spectrum includes necrotizing acute inflammation, relatively non-inflammatory haemorrhage into the airspaces with focal acellular necrosis, and coagulation necrosis with necrotic pulmonary muscular veins and arteries [35]. Several lines of evidence suggest that the vasculitis and septic infarcts occur predominantly in pneumonia that is secondary to bacteraemia [35]. Of these hallmark features, non-inflammatory haemorrhage into the airspaces was not a marked feature of the histopathology present in our sheep, neither was it apparently a feature of the original rat model described by Cash et al (1979) [24]. These authors also described changes in the bronchial mucosa, involving goblet cell hyperplasia and metaplasia, and bronchiectasis.

Our interest in modelling *Pseudomonas* infection in sheep stems from the desire to develop systems that mimic pathology associated with chronic human infection – particularly in the context of cystic fibrosis. Thus, we sought to determine whether the noted responses held any parallels with pathology seen in patients with cystic fibrosis. Baltimore, Christie and Smith (1989) [36] described the relationship between *P. aeruginosa* organisms and surrounding pathology in autopsy lung specimens derived from patients with a diagnosis of cystic fibrosis. These authors described changes present in larger (>1 mm diameter) and smaller (<1 mm) airways [36]. The larger airways were frequently plugged with exudate consisting almost entirely of neutrophils and sequestered microcolonies of *Pseudomonas*. There were erosions of the epithelium in the airways and *Pseudomonas* were attached to the denuded membrane or inflamed and granulating surface. In the smaller airways there was often focal or total loss of bronchiolar epithelium. Exudate plugs were associated with central bacterial clusters or peripherally dispersed organisms. In airways lacking epithelium, the tendency was for the lumen to fill in from the outside to the centre with proliferation of fibrous tissue in a granulation tissue response; in some instances, there appeared to be preservation of lumen size, but with mural thickening by granulation tissue and centrifugal spread of inflammation into surrounding alveolar parenchyma. The granulation tissue response surrounded the intra-luminal bacteria and was not invaded by them. Tissue that had progressed to fibrosis consistently lacked visible organisms. The observed centrifugal spread of a granulation response away from a central bacterial presence holds similarity with our observation of some granulomas being circumscribed by circumferential rings of fibroblasts and collagen, consistent with fibroplasia and fibrosis.

The pathophysiology of cystic fibrosis lung disease develops as a consequence of mutations in the cystic fibrosis transmembrane conductance regulator (CFTR) protein which is normally expressed in the airways and distal lung. Compromised function of this ion channel leads to dehydrated airway surfaces lined with thickened secretions and mucus accumulations. This environment serves as a nidus for bacterial infection. The agar bead approach can only attempt to model the effect of chronic localisation of *Pseudomonas* in the lung and does not mimic the fundamental pathophysiology of CF. Notable recent advances in this regard have been the targeted inactivation of the CFTR gene in the ferret [37] and pig [38] which has resulted in CF models that, in comparison with available mutants in mice, more closely resembles the progression of human CF pathology. The further development of these and other models across species will be crucial to understanding CF pathophysiology (including the interplay between CFTR function and bacterial infection), and developing effective therapies for this disease.

No clinical evidence of systemic or organ compromise developed in the sheep during infection. This contrasts with mice where, in addition to marked weight loss, the extensive neutrophil influx that occurs in response to the P. *aeruginosa*-laden beads often causes airway obstruction, compromised gas exchange and significant mortality [39]. Whilst weight loss also occurs in the rat model [27], mortality is generally unexpected [27], [40]. In only targeting a small proportion of the whole lung substance, segmental approaches in larger animals intuitively lower the potential for respiratory compromise arising as a consequence of the insult, and thus offer some ethical advantages over related studies in smaller species.

Applying different protocols to different segments within the same lung offers the potential of using certain segments as ‘within-lung’ controls, thus reducing the confounding variability associated with biological variability between animals. The validity of this approach needs careful consideration in every context for which it is proposed – particularly in instances where active lung infection is a feature of the model system. In this model, whilst there was no evidence of infection present in BALF from control segments, *Pseudomonas* infection was found in tissue samples from four control segments in three sheep, out of a total of thirty samples derived from control, or agar-only instilled, segments. The lack of evidence for infection in the bronchoalveolar space in these control segments, and the association with substantial pleural involvement in the neighbouring lobe (RC) in two of the circumstances implies the potential for contiguous spread via this (pleural) route. In a monograph on the interrelationship of pleural and pulmonary interstitial liquid, Wiener-Kronish and Broaddus (1993) [41] refer to unpublished observations in which lung instillation of live *P*.*aeruginosa* was associated with increased permeability across the alveolar capillary membrane such that protein tracers tracked from airspace to bloodstream and vice versa. Of particular significance to the current study was the observed formation of protein-rich pleural effusions which were deemed to derive from the airspaces on account of the presence of bacteria and protein tracer from that compartment. Certainly the histological evidence in the sheep of interlobular septal expansion extending to the pleural surface suggests the potential for translocation of bacteria, or bacteria-laden cells, through pleurolymphatic communications. These observations indicate that where there is a defined and critical need to compare *Pseudomonas*-induced responses against a true negative control, then those comparisons should be made on a between-animal procedure-control basis.

We observed some within-animal variation in the response of different lung segments to the same infection challenge. Such variation is consistent with the emerging appreciation that there is marked regional heterogeneity across the lung on several levels. Indeed, in young adults with stable CF, and in young children with CF experiencing exacerbations there is regional heterogeneity of lung inflammation [42], [43], in patients with COPD there are also important regional differences in secretory IgA associated with the site and nature of airway remodelling, inflammation and potential susceptibility to infection [44], and it is recognised that there are regional differences in the local responses to stress within the lung [45]. Understanding and quantifying the extent of such variability is a necessary prerequisite to the design of appropriately powered segmental challenge experiments in future.

Whilst, in sheep DR18, only one of five originally infected segments still had evidence of tissue infection at day 66 there was histological evidence of abnormality in all the previously infected segments – with beads still serving as foci for the pyogranulomatous pneumonia. Given that, under pressure from antibiotics, a very small proportion of *P. aeruginosa* persister cells survive antibiotic exposure by going into a slow-growing dormant state [46] it is worth speculating whether a similar phenomenon could be operating *in vivo* in this model. The lack of bacterial growth may reflect adaptation under the pressure brought about by innate and adaptive immunity that still responds to the presence of antigen in the bead matrices.

The trend towards an increase in the relative proportion of lymphocytes detected in BAL fluid as time after initial infection extended beyond d10 is of some interest, and gels with the histological evidence of a lymphocyte predominance in the sheep infected for 66 days. Whilst cystic fibrosis is characterised by a neutrophil-dominated inflammation present in the airway lumen, the bronchial mucosa is characterised by the recruitment and accumulation of lymphocytes [47], [48]. Certainly BALT hyperplasia was a noted feature of the response to *Pseudomonas* beads instillation in the sheep model and the shift in bias from the early neutrophil response towards the later lymphocytic response perhaps suggests that the successful host response towards *Pseudomonas*, evidenced by the fall in tissue bacterial burden, requires the inflammatory influx and eventual migration of lymphocytes into the airspaces.

This study also provided the opportunity to assess the sensitivity and specificity of quantitative cultures of BAL fluid in detecting the presence of *Pseudomonas* infection in this model. The levels of infection detectable in BAL fluid were generally very low and contrasted with higher levels of infection measured in tissue. It is of interest to contrast these observations with studies aimed at determining the association between quantitative culture of lower respiratory tract secretions and the diagnosis of ventilator associated pneumonia (VAP) in patients. In a benchmark study involving the immediate post-mortem sampling of lung tissue from still-ventilated patients Chastre et al (1984) [49] established that, in six patients with pneumonia confirmed by histologic criteria, all had at least one microorganism obtained at a concentration of >10^4^ cfu/g of lung tissue. This, and a subsequent study by Chastre and colleagues [50], demonstrated a good correlation between lung tissue cultures and protected specimen brush (PSB) and BAL cultures in patients without prior antibiotic treatment and these studies have helped to establish 10^3^ and 10^4^ cfu/ml as the thresholds to distinguish colonization from alveolar infection in PSB and BAL sample cultures respectively. If we applied such criteria to this model, using the presence of >10^4^ *Pseudomonas* cfu/g of lung tissue as the gold standard indicator of infection, then the sensitivity of BAL sample culture would only be 5% and the specificity 36%. Even relaxing the threshold to simply identifying the presence of ≥1 cfu/g only raises the sensitivity to 75% and the specificity falls to 18%. Quantitative culture of BAL fluid is therefore an insensitive method to detect the presence of tissue infection in this model. Whether the nature of the model, in deliberately lodging beads in the airspaces and allowing colony growth to proceed within the confines of the agar environment, restricts the ability of lavage fluid to reflect the actual bacterial burden in the distal lung must remain conjectural but does raise the issue of relevance to chronic *Pseudomonas* infection in patients, where *Pseudomonas* organisms may similarly find their niche in a protective biofilm and prove potentially difficult to sample by BAL. Whilst there appears to be a paucity of data in the literature with which to compare BAL fluid and tissue bacterial burdens in the *Pseudomonas* bead model, one recent study in mice examined both compartments and determined that 7–14 days after inoculation of agar beads containing 5×10^7^ *P. aeruginosa* the bacterial load in lung tissue was approximately 1×10^5^ cfu/g lung tissue, whereas only 33% of those mice were positive on culture of BAL fluid [51] – a finding that reflects our experience using the sheep segmental model. Notably, in a recently developed large animal model of experimental pneumonia induced by the inoculation of a suspension of 10^6^ cfu *P*. *aeruginosa* in ventilated piglets [52], BAL cultures obtained 96 h after inoculation in five piglets yielded *P. aeruginosa* in a concentration >10^4^ cfu/ml in four, and >10^5^ cfu/ml in the remaining piglet. The associated lung tissue cultures showed growth of *P. aeruginosa* in a concentration >10^4^ cfu/g in all the samples evaluated.

In summary, we have demonstrated in this study that endobronchial instillation of sheep with *Pseudomonas*-laden agar beads can lead to the development of a chronic, suppurative, necrotising and pyogranulomatous pneumonia centred on the instilled beads. Whilst in the majority of instances infection remains localised at the point of instillation, on the occasional instance there was transfer to neighbouring areas of the lung. Following an initial predominant neutrophilic response the burden of infection decreased over time but was still apparent in some segments 66 days after initial infection. Sheep exhibited neither overt evidence of systemic malaise, nor evidence of respiratory compromise during infection. Whilst histological features of the ovine bead model of chronic *Pseudomonas* infection are consistent with those previously documented using the same model in other species and with the major features of *Pseudomonas* infection in patients with cystic fibrosis the predominant localisation of pathology in the distal lung parenchyma is at variance with the early bronchiolar localisation of inflammation in the CF lung, and does not therefore mimic the pathophysiology of CF. Quantitative culture of BAL fluid proved poorly sensitive in detecting clinically relevant levels of infection (>10^4^ cfu/g) in lung tissue.

The benefits of using large animals as models for respiratory disease relate to the closer anatomic and physiologic relevance of these systems to the human lung, and their largely faithful reproduction of the principal pathophysiological elements of human lung disease. In addition, such systems facilitate the application of a variety of bronchoscopic and imaging modalities that bear immediate relevance to their clinical usage, and hence can be considered truly translational in that sense. Lastly, the adoption of a segmental approach to lung infection carries significant ethical advantage in that the animal suffers no obvious clinical compromise and provides a mechanism whereby differential treatments may be evaluated within the same animal over the same course of time. The potential value of this system relates to both understanding the immune and inflammatory mechanisms that underlay such lung infections, and their resolution, as well as in providing a translational platform to evaluate novel therapeutic approaches, for which there is widely recognised need at the present time.

## Acknowledgments

The authors wish to acknowledge the assistance of Dryden Animal Services in the conduct of this work and Juan Manuel Cárdenas Maestre (Department of Chemistry, University of Edinburgh) for the bead sizing.

## References

[1] FitzSimmons SC (1993) The changing epidemiology of cystic fibrosis. J Pediatr 122: 1–9. 8419592 10.1016/s0022-3476(05)83478-x

[2] Burns JL, Gibson RL, McNamara S, Yim D, Emerson J, et al (2001) Longitudinal assessment of Pseudomonas aeruginosa in young children with cystic fibrosis. J Infect Dis 183: 444–452. 11133376 10.1086/318075

[3] Govan JR, Deretic V (1996) Microbial pathogenesis in cystic fibrosis: mucoid Pseudomonas aeruginosa and Burkholderia cepacia. Microbiol Rev 60: 539–574. 8840786 10.1128/mr.60.3.539-574.1996 PMC239456

[4] Hurley MN, Cámara M, Smyth AR (2012) Novel approaches to the treatment of Pseudomonas aeruginosa infections in cystic fibrosis. The European respiratory journal: official journal of the European Society for Clinical Respiratory Physiology 40: 1014–1023. 10.1183/09031936.00042012 PMC3461346 22743672

[5] Ballmann M, Smyth A, Geller DE (2011) Therapeutic approaches to chronic cystic fibrosis respiratory infections with available, emerging aerosolized antibiotics. Respir Med. England: 2011 Elsevier Ltd. S2–8. 10.1016/S0954-6111(11)70021-X 22208546

[6] Geller DE, Flume PA, Staab D, Fischer R, Loutit JS, et al (2011) Levofloxacin inhalation solution (MP-376) in patients with cystic fibrosis with. Am J Respir Crit Care Med 183: 1510–1516. 21471106 10.1164/rccm.201008-1293OC

[7] McCoy KS, Quittner AL, Oermann CM, Gibson RL, Retsch-Bogart GZ, et al (2008) Inhaled aztreonam lysine for chronic airway Pseudomonas aeruginosa in cystic. Am J Respir Crit Care Med 178: 921–928. 18658109 10.1164/rccm.200712-1804OC PMC2577727

[8] Emerson M, Renwick L, Tate S, Rhind S, Milne E, et al (2003) Transfection efficiency and toxicity following delivery of naked plasmid DNA and cationic lipid-DNA complexes to ovine lung segments. MolTher 8: 646–653. 10.1016/s1525-0016(03)00233-8 14529838

[9] McLachlan G, Baker A, Tennant P, Gordon C, Vrettou C, et al (2007) Optimizing aerosol gene delivery and expression in the ovine lung. MolTher 15: 348–354. 10.1038/sj.mt.6300058 17235313

[10] McLachlan G, Davidson H, Holder E, Davies LA, Pringle IA, et al (2011) Pre-clinical evaluation of three non-viral gene transfer agents for cystic fibrosis after aerosol delivery to the ovine lung. Gene Ther 18: 996–1005. 21512505 10.1038/gt.2011.55

[11] Pringle IA, McLachlan G, Collie DD, Sumner-Jones SG, Lawton AE, et al (2007) Electroporation enhances reporter gene expression following delivery of naked plasmid DNA to the lung. JGene Med 9: 369–380. 17410613 10.1002/jgm.1026

[12] Rejman J, De Fino I, Paroni M, Bragonzi A, Demeester J, et al (2010) Impact of chronic pulmonary infection with Pseudomonas aeruginosa on transfection mediated by viral and nonviral vectors. Hum Gene Ther 21: 351–356. 19788388 10.1089/hum.2009.085

[13] van Heeckeren A, Ferkol T, Tosi M (1998) Effects of bronchopulmonary inflammation induced by pseudomonas aeruginosa on adenovirus-mediated gene transfer to airway epithelial cells in mice. Gene Ther 5: 345–351. 9614554 10.1038/sj.gt.3300593

[14] Chang AB, Faoagali J, Cox NC, Marchant JM, Dean B, et al (2006) A bronchoscopic scoring system for airway secretions – airway cellularity and microbiological validation. PediatrPulmonol 41: 887–892. 10.1002/ppul.20478 16858700

[15] Govan JR, Fyfe JA (1978) Mucoid Pseudomonas aeruginosa and cystic fibrosis: resistance of the mucoid from to carbenicillin, flucloxacillin and tobramycin and the isolation of mucoid variants in vitro. J Antimicrob Chemother 4: 233–240. 97259 10.1093/jac/4.3.233

[16] Capuano SV, Croix DA, Pawar S, Zinovik A, Myers A, et al (2003) Experimental Mycobacterium tuberculosis infection of cynomolgus macaques closely resembles the various manifestations of human M. tuberculosis infection. Infect Immun 71: 5831–5844. 14500505 10.1128/IAI.71.10.5831-5844.2003 PMC201048

[17] Lin PL, Rodgers M, Smith L, Bigbee M, Myers A, et al.. (2009) Quantitative comparison of active and latent tuberculosis in the cynomolgus macaque model. Infect Immun. United States. 4631–4642. 10.1128/IAI.00592-09 PMC2747916 19620341

[18] Olsen RJ, Ashraf M, Gonulal VE, Ayeras AA, Cantu C, et al (2010) Lower respiratory tract infection in cynomolgus macaques (Macaca fascicularis) infected with group A Streptococcus. Microb Pathog 49: 336–347. 20674736 10.1016/j.micpath.2010.06.012

[19] Jassal MS, Nedeltchev GG, Osborne J, Bishai WR (2011) A modified scoring system to describe gross pathology in the rabbit model of tuberculosis. BMC Microbiol 11: 49. 21375756 10.1186/1471-2180-11-49 PMC3058006

[20] Reinhold P, Ostermann C, Liebler-Tenorio E, Berndt A, Vogel A, et al (2012) A bovine model of respiratory Chlamydia psittaci infection: challenge dose titration. PLoS One 7: e30125. 22299031 10.1371/journal.pone.0030125 PMC3267716

[21] Grubor B, Gallup JM, Ramirez-Romero R, Bailey TB, Crouch EC, et al (2004) Surfactant protein D expression in normal and pneumonic ovine lung. Veterinary Immunology and Immunopathology 101: 235–242. 15350753 10.1016/j.vetimm.2004.05.004

[22] Brogden KA, Kalfa VC, Ackermann MR, Palmquist DE, McCray PB, et al (2001) The ovine cathelicidin SMAP29 kills ovine respiratory pathogens in vitro and in an ovine model of pulmonary infection. Antimicrob Agents Chemother 45: 331–334. 11120991 10.1128/AAC.45.1.331-334.2001 PMC90286

[23] Patterson GA, Todd TR (1982) A large animal model of pseudomonas pneumonia. J Surg Res 33: 214–219. 7109568 10.1016/0022-4804(82)90032-4

[24] Cash HA, Woods DE, McCullough B, Johanson WG, Bass JA (1979) A rat model of chronic respiratory infection with Pseudomonas aeruginosa. Am Rev Respir Dis 119: 453–459. 109021 10.1164/arrd.1979.119.3.453

[25] Alhariri M, Omri A (2013) Efficacy of Liposomal Bismuth-Ethanedithiol-Loaded Tobramycin after Intratracheal Administration in Rats with Pulmonary Pseudomonas aeruginosa Infection. Antimicrob Agents Chemother 57: 569–578. 23147741 10.1128/AAC.01634-12 PMC3535983

[26] Growcott EJ, Coulthard A, Amison R, Hardaker EL, Saxena V, et al (2011) Characterisation of a refined rat model of respiratory infection with Pseudomonas aeruginosa and the effect of ciprofloxacin. J Cyst Fibros 10: 166–174. 21247812 10.1016/j.jcf.2010.12.007

[27] Hraiech S, Brégeon F, Brunel JM, Rolain JM, Lepidi H, et al (2012) Antibacterial efficacy of inhaled squalamine in a rat model of chronic Pseudomonas aeruginosa pneumonia. J Antimicrob Chemother 67: 2452–2458. 22744759 10.1093/jac/dks230

[28] Meers P, Neville M, Malinin V, Scotto AW, Sardaryan G, et al (2008) Biofilm penetration, triggered release and in vivo activity of inhaled liposomal amikacin in chronic Pseudomonas aeruginosa lung infections. J Antimicrob Chemother 61: 859–868. 18305202 10.1093/jac/dkn059

[29] Nam SW, Chen X, Lim J, Kim SH, Kim ST, et al (2011) In vivo fluorescence imaging of bacteriogenic cyanide in the lungs of live mice infected with cystic fibrosis pathogens. PLoS One 6: e21387. 21750709 10.1371/journal.pone.0021387 PMC3131278

[30] Dagenais A, Gosselin D, Guilbault C, Radzioch D, Berthiaume Y (2005) Modulation of epithelial sodium channel (ENaC) expression in mouse lung infected with Pseudomonas aeruginosa. Respir Res 6: 2. 15636635 10.1186/1465-9921-6-2 PMC546414

[31] Thomassen MJ, Klinger JD, Winnie GB, Wood RE, Burtner C, et al (1984) Pulmonary cellular response to chronic irritation and chronic Pseudomonas aeruginosa pneumonia in cats. InfectImmun 45: 741–747. 10.1128/iai.45.3.741-747.1984 PMC263360 6432697

[32] Winnie GB, Klinger JD, Sherman JM, Thomassen MJ (1982) Induction of phagocytic inhibitory activity in cats with chronic Pseudomonas aeruginosa pulmonary infection. Infect Immun 38: 1088–1093. 6818144 10.1128/iai.38.3.1088-1093.1982 PMC347861

[33] Cheung AT, Moss RB, Leong AB, Novick WJ (1992) Chronic Pseudomonas aeruginosa endobronchitis in rhesus monkeys: I. Effects of pentoxifylline on neutrophil influx. J Med Primatol 21: 357–362. 1307753

[34] Cheung AT, Moss RB, Kurland G, Leong AB, Novick WJ (1993) Chronic Pseudomonas aeruginosa endobronchitis in rhesus monkeys: II. A histopathologic analysis. J Med Primatol 22: 257–262. 8230177

[35] Tomashefski JT, Dail DH (2008) Dail and Hammar's Pulmonary Pathology: Nonneoplastic lung disease: Springer. 1301 p.

[36] Baltimore RS, Christie CD, Smith GJ (1989) Immunohistopathologic localization of Pseudomonas aeruginosa in lungs from patients with cystic fibrosis. Implications for the pathogenesis of progressive lung deterioration. Am Rev Respir Dis 140: 1650–1661. 2513765 10.1164/ajrccm/140.6.1650

[37] Sun X, Yan Z, Yi Y, Li Z, Lei D, et al.. (2008) Adeno-associated virus-targeted disruption of the CFTR gene in cloned ferrets. JClinInvest. 10.1172/JCI34599 PMC2263147 18324338

[38] Rogers CS, Hao Y, Rokhlina T, Samuel M, Stoltz DA, et al.. (2008) Production of CFTR-null and CFTR-DeltaF508 heterozygous pigs by adeno-associated virus-mediated gene targeting and somatic cell nuclear transfer. JClinInvest. 10.1172/JCI34773 PMC2265103 18324337

[39] van Heeckeren AM, Schluchter MD (2002) Murine models of chronic Pseudomonas aeruginosa lung infection. Lab Anim 36: 291–312. 12144741 10.1258/002367702320162405

[40] Kukavica-Ibrulj I, Bragonzi A, Paroni M, Winstanley C, Sanschagrin F, et al (2008) In vivo growth of Pseudomonas aeruginosa strains PAO1 and PA14 and the hypervirulent strain LESB58 in a rat model of chronic lung infection. J Bacteriol 190: 2804–2813. 18083816 10.1128/JB.01572-07 PMC2293253

[41] Wiener-Kronish JP, Broaddus VC (1993) Interrelationship of pleural and pulmonary interstitial liquid. Annu Rev Physiol 55: 209–226. 8466174 10.1146/annurev.ph.55.030193.001233

[42] Meyer KC, Sharma A (1997) Regional variability of lung inflammation in cystic fibrosis. Am J Respir Crit Care Med 156: 1536–1540. 9372672 10.1164/ajrccm.156.5.9701098

[43] Davis SD, Fordham LA, Brody AS, Noah TL, Retsch-Bogart GZ, et al.. (2007) Computed tomography reflects lower airway inflammation and tracks changes in early cystic fibrosis. Am J Respir Crit Care Med. United States. 943–950. 10.1164/rccm.200603-343OC 17303797

[44] Polosukhin VV, Cates JM, Lawson WE, Zaynagetdinov R, Milstone AP, et al.. (2011) Bronchial secretory immunoglobulin a deficiency correlates with airway inflammation and progression of chronic obstructive pulmonary disease. Am J Respir Crit Care Med. United States. 317–327. 10.1164/rccm.201010-1629OC PMC3265275 21512171

[45] Simon BA, Easley RB, Grigoryev DN, Ma SF, Ye SQ, et al (2006) Microarray analysis of regional cellular responses to local mechanical stress in acute lung injury. AmJPhysiol Lung Cell MolPhysiol 291: L851–L861. 10.1152/ajplung.00463.2005 16782753

[46] Lewis K (2010) Persister cells. Annu Rev Microbiol 64: 357–372. 20528688 10.1146/annurev.micro.112408.134306

[47] Regamey N, Tsartsali L, Hilliard TN, Fuchs O, Tan HL, et al (2012) Distinct patterns of inflammation in the airway lumen and bronchial mucosa of children with cystic fibrosis. Thorax 67: 164–170. 22008188 10.1136/thoraxjnl-2011-200585

[48] Hubeau C, Lorenzato M, Couetil JP, Hubert D, Dusser D, et al.. (2001) Quantitative analysis of inflammatory cells infiltrating the cystic fibrosis airway mucosa. Clin Exp Immunol. England. 69–76. 10.1046/j.1365-2249.2001.01456.x PMC1906034 11359444

[49] Chastre J, Viau F, Brun P, Pierre J, Dauge MC, et al (1984) Prospective evaluation of the protected specimen brush for the diagnosis of pulmonary infections in ventilated patients. Am Rev Respir Dis 130: 924–929. 6497170 10.1164/arrd.1984.130.5.924

[50] Chastre J, Fagon JY, Bornet-Lecso M, Calvat S, Dombret MC, et al (1995) Evaluation of bronchoscopic techniques for the diagnosis of nosocomial pneumonia. Am J Respir Crit Care Med 152: 231–240. 7599829 10.1164/ajrccm.152.1.7599829

[51] Ding FM, Zhu SL, Shen C, Jiang YQ (2012) Low-dose clarithromycin therapy modulates CD4(+) T-cell responses in a mouse model of chronic Pseudomonas aeruginosa lung infection. Respirology 17: 727–734. 22404374 10.1111/j.1440-1843.2012.02166.x

[52] Sibila O, Agusti C, Torres A, Baquero S, Gando S, et al.. (2007) Experimental Pseudomonas aeruginosa pneumonia: evaluation of the associated inflammatory response. Eur Respir J. Switzerland. 1167–1172. 10.1183/09031936.00053607 17804447

